# Role of *Bifidobacterium* in Modulating the Intestinal Epithelial Tight Junction Barrier: Current Knowledge and Perspectives

**DOI:** 10.1016/j.cdnut.2023.102026

**Published:** 2023-10-30

**Authors:** Raz Abdulqadir, Jessica Engers, Rana Al-Sadi

**Affiliations:** Penn State College of Medicine, Hershey Medical Center, Hershey, PA, United States

**Keywords:** intestinal tight junction barrier, gastrointestinal inflammation, Bifidobacterium, cytokines, gut microbiota

## Abstract

The intestinal tight junction (TJ) barrier is a crucial defense mechanism that prevents the passage of intestinal content into the intestinal wall, tissue, and systemic circulation. A compromised intestinal TJ barrier has been identified as a significant factor in inflammatory bowel disease (IBD), necrotizing enterocolitis, and other gut-related inflammatory conditions. Recent studies have revealed the importance of the probiotic bacterial strains of *Bifidobacterium* in protecting against intestinal inflammation and IBD pathogenesis via the regulation of intestinal TJ barrier function. Numerous species and strains of *Bifidobacterium* have been found to regulate TJ proteins and the signaling pathways responsible for maintaining intestinal barrier integrity and permeability. In this review, we provide a summary of recent studies that highlight the regulatory role of *Bifidobacterium* species and the strain effect on the intestinal TJ barrier. We also discuss the intracellular mechanisms involved in *Bifidobacterium* modulation of the intestinal barrier and the potential therapeutic efficacy of targeting the barrier function to regulate intestinal inflammation.

## Introduction

**FIGURE 1:**
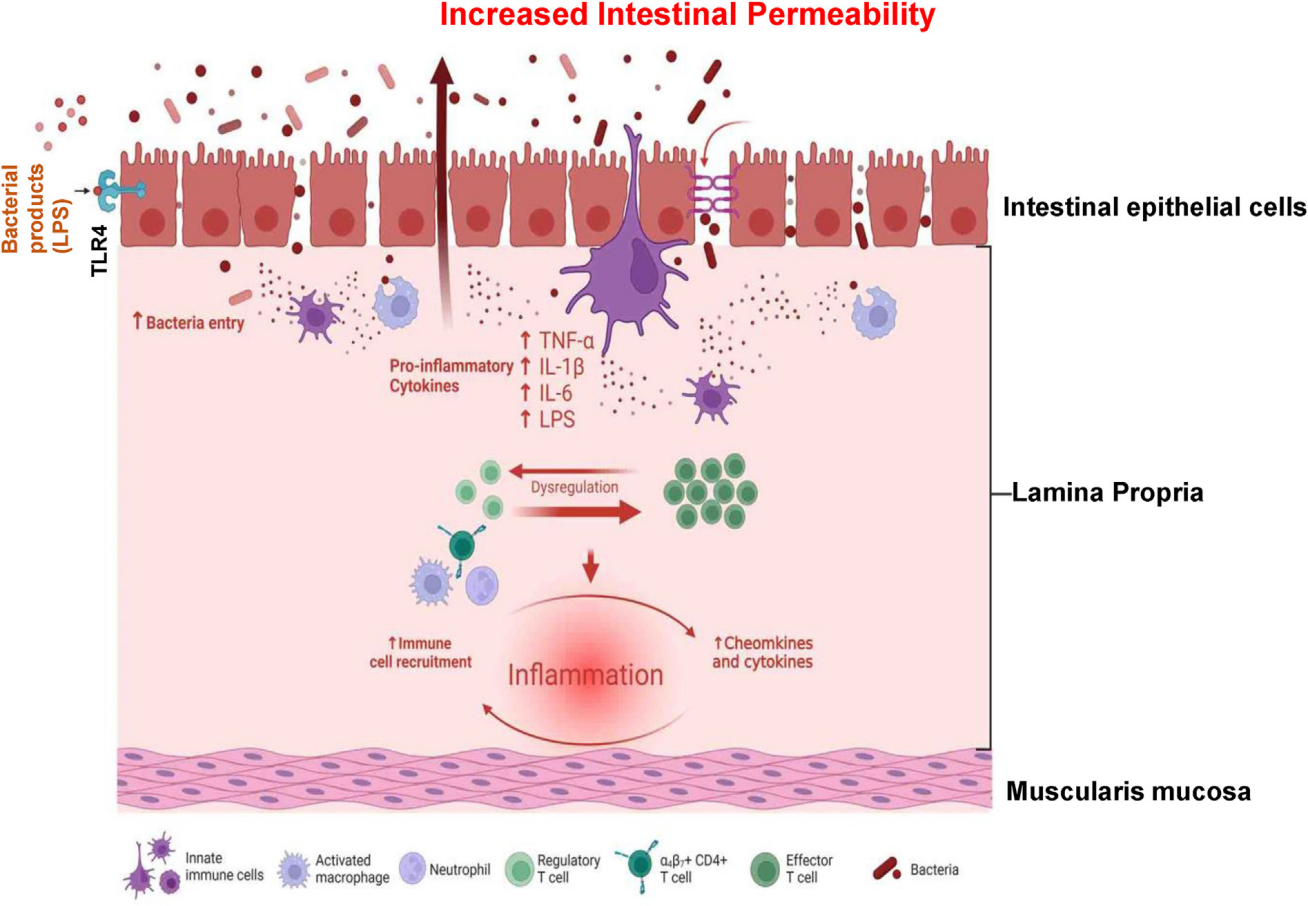
The impaired tight junction barrier plays a crucial role in the pathology of inflammatory bowel disease. When this barrier is compromised within the intestinal tract, it facilitates the passage of antigens and large molecules such as bacteria and their byproducts into the lamina propria. This breach in intestinal integrity subsequently leads to increased intestinal permeability and triggers an uncontrolled and dysregulated inflammatory response. Abbreviation: TLR, toll-like receptor. (Created with BioRender).

The intestinal epithelium cells (IECs) act as a critical barrier, separating luminal contents from internal tissues. These cells maintain selective permeability by regulating the transport of molecules through tight junctions (TJs), which serve as structural and functional barriers against the passage of luminal substances. Cell junctions in IECs have a crucial role in facilitating selective paracellular permeability. This selective permeability allows for the absorption of essential nutrients, regulates the transportation of ions and macromolecules, and provides protection to IECs against various toxins (Figure 1). The TJ complex is composed of occludin and claudin proteins, adhesion junctions (integral membrane-bound cadherins and cytoplasmic accessory proteins, i.e., α- and β-catenin), junctional adhesion molecule A (JAM-A), and accessory proteins (zonula occludens [ZO]-1, ZO-2, ZO-3) [[1], [2], [3], [4]]. These TJs play a crucial role in preserving epithelial polarity and ensuring the proper functioning of the paracellular barrier as a physical barrier against luminal content including microorganisms, antigens, and xenobiotics [1,[5], [6], [7]]. Figure 1 illustrates the development and critical impact of an impared intestinal epithelial barrier. Accumulating evidence indicates that TJ opening is triggered by the phosphorylation of myosin light chain, which predominantly depends on myosin light-chain kinase (MLCK) activation. This makes MLCK a key regulator of intestinal TJ barrier function [8,9]. The defective intestinal TJ barrier, also known as “leaky gut,” significantly contributes to the development of several chronic inflammatory conditions [6,7]. These conditions include celiac disease, inflammatory bowel disease (IBD) [10,11], necrotizing enterocolitis (NEC) [[12], [13], [14]], alcoholic liver disease [[15], [16], [17]], fatty liver disease [18,19], and rheumatoid arthritis (RA) [[20], [21], [22], [23]].

Therapeutic tightening of the intestinal TJ barrier could be an important treatment option for IBD and other inflammatory gut conditions [24]. Recent studies have emphasized the vital role of probiotics in cellular functions, such as regulating TJs and adaptor proteins, as well as maintaining the intestinal barrier. Moreover, an expanding body of research highlights the advantages of targeting the gut microbiota in the treatment of IBD [25,26]. Based on systematic review and meta-analysis studies, probiotics have shown substantial evidence of enhancing gut barrier function, reducing inflammation, and modulating the gut microbiota structure. These benefits are achieved primarily through the enrichment and colonization of *Lactobacillus* and *Bifidobacterium* species [27]. Additional studies have demonstrated that the administration of different probiotic strains, mainly *Bifidobacteria*, can directly influence and regulate both the TJ barrier function and the host immune response by modulating different signaling pathways in IECs (Table 1) [[28], [29], [30], [31], [32], [33], [34], [35], [36], [37], [38], [39], [40], [41], [42], [43], [44], [45], [46], [47], [48], [49], [50], [51], [52], [53], [54], [55], [56], [57], [58]].

**TABLE 1:** *Bifidobacteria* species and strains have the potential to regulate the TJ barrier in vitro or in a mouse model of colitis, with a specific focus on targeting the function of the TJ barrier

Probiotic bacteria	Cell and/or animal model	Treatment or disease condition	Effect on TJ proteins and barrier function	Mode of mechanism and effect on animal model	References
*Bifidobacterium (B.)*	Caco-2; Rat NEC	LPS damage	Restore occludin, claudin-3, ZO-1	Normalize intestinal permeability, TNF-α, IL-6.	[28]
*Bifidobacterium (BIF)*	Caco-2	TNF-α induced inflammation	Increase TER	Suppress TNF-α-induced overexpression of IL-6, IL-8, and autophagy pathway.	[29]
*B. adolescentis* CGMCC15058	Rat	D-galactosamine induced liver injury	Enhance ZO-1 expression	Decrease the proinflammatory cytokine TNF-α and IL-6 and enhance mucin-4 expression.	[30]
*B. adolescentis* IM38	Caco-2; Mouse	HFD, LPS damage, colitis	Enhance ZO-1, occludin and claudin-1 expression	Inhibit NF-κB activation; increase HFD suppressed expression of IL-10).	[31]
*B. animalis* ssp. lactis CNCM-I2494	Mouse model of low-grade inflammation	Chronic DNBS-induced low-grade inflammation	Restore intestinal permeability and normalize claudin-4	Decrease the proinflammatory cytokines IL-12, IL-2, IL-4 and IFN-γ.	[32]
*B. bifidium*	Rat	NEC	Normalize occludin and claudin-3 expression and localization the ileum	Normalize levels of IL-6, mucin-3, and Tff3 in the ileum.	[33]
*B. bifidium* (BB1)	Caco-2; Mouse	DSS-induced colitis	Increase TER, decrease intestinal permeability	Activation of TLR2, p38 kinase activity, and restore intestinal permeability.	[34]
*B. bifidium* (BB1)	Caco-2	—	Increase TER, decrease inulin flux	Enhances intestinal epithelial TJ barrier in a strain-specific and TLR2/TOLLIP dependent manner, independent of MYD88 pathway.	[35]
*B. bifidium* (BB1)	Caco-2; Mouse	IL-1β induced inflammation	Increase TER, decrease inulin; decrease intestinal permeability	Prevent the IL-1β induced increase in intestinal permeability by TLR2 dependent activation of PPAR-γ and inhibition of NF-κB and MLCK signaling pathway.	[36]
*B. bifidium E3 and B. longum subsp. infantis E4*	Mouse	LPS-induced intestinal injury	Increase ZO-1, occludin, claudin-1	Ameliorate LPS-induced injury by enhancing intestinal TJ barrier and intestinal mucosal immunity, reducing intestinal inflammation (IL-1β, IL-6, and TNF-α) by inhibiting TLR4/NF-κB MAPK signaling pathways.	[37]
*B. bifidium* FL-228.1	Caco-2; Mouse	DSS-induced colitis	Increase TER, decrease permeability, and increase claudin-4	Protects against DSS-induced intestinal barrier damage by increasing ratio of IL-10/IL-12, increase mucin-2, inhibits NLRP3, and activates PPAR-γ.	[38]
*B. bifidium* FL-228.1 and B. bifidium FL-276.1	Caco-2; Mouse	DSS-induced colitis	Increase TER, decrease permeability, and increase ZO-1, occludin, and claudin-4	Protects against DSS-induced intestinal barrier damage by increasing production of indole-3-lactic acid by activating AHR/NRF2/NLRP3 inflammasome pathways.	[39]
*B. breve* CCFM1078	Rat	collagen-induced arthritis	Normalize ZO-1, claudin-1, claudin-3, occludin, and JAM-B	Alleviates collagen-induced arthritis, repairs damage to the intestinal barrier, and modulates the gut microbiota through the inhibition of TLR4/MYD88-dependent pathways.	[40]
*B. dentium N8, isolated from the feces of breastfed infants*	Caco-2	LPS	Increases ZO-1, occludin, claudin-1 mRNA expression/downregulated mRNA expression level of TLR4 and proinflammatory cytokines (TNF-α, IL1B, IL6). Also increases TER and decreases paracellular (FITC) permeability of LPS-stimulated Caco-2	Anti-inflammation (downregulate mRNA expression level of TLR4 and proinflammatory cytokines) (TNF-α, IL-1β, IL-6).	[41]
*B. infantis*	Neonatal mouse	NEC	Preserve intestinal permeability and normalize claudin-4 and occludin	—	[42]
*B. infantis* 35624	Mouse	—	Increase colonic TER	Inhibition of colonic ion transport.	[43]
*B. infantis* conditioned medium	Caco-2, H4 cells	IL-1β induced damage	Increase TER and occludin, decrease claudin-1	Inhibit NF-κB p65 activation.	[44]
*B. infantis* conditioned media	T84 human epithelial cells; IL-10-deficient mouse	TNF-α and IFN-γ damage	Increase TER; increase ZO-1 and occludin expression and decrease claudin-2 expression	Attenuate inflammation, normalize colonic permeability, and decrease colonic and splenic IFN-γ secretion.	[45]
*B. lactis* 420	Caco-2	—	Increase TER	—	[46]
*B. longum* CCM 7952	HEK293; Mouse	DSS-induced colitis	Decrease colon permeability; restore ZO-1 and occludin expression	Activation of TLR2 and NOD2 pathways.	[47]
*B. longum* HB55020	Mouse	*Trichinella spiralis* infection induced IBS	No effect on TER; restore ZO-1 and claudin-1 expression	Change gut microbiota.	[48]
*B. longum* LC67	Mouse	TNBS-induced colitis	Prevent TNBS-suppressed expression of ZO-1, occludin and claudin-1	Prevent TNBS-induced NF-κB activation and restore Th17/Treg balance.	[49]
*B. longum* LC67	Caco-2, Mouse	Ethanol and LPS damage, alcohol steatosis	Increase the ethanol/LPS-suppressed occludin and claudin-1	Reduce ethanol-induced ALT, AST, TG, and TC levels in the blood and liver and inhibit NF-κB activation.	[50]
*B. longum* subsp. *longum* 2-21-4	CCD841 CoN	LPS damage	Restore ZO-1, claudin-1, and occludin	Prevent LPS-induced intestinal barrier injury, enhance cell proliferation, reduce apoptosis, and active Wnt/β-catenin and PI3K/Akt/mTOR signaling pathway.	[51]
*B. longum* subsp. *longum* K5 and K15	HT-29, Caco-2 RAW264.7	LPS damage	Increase TER, increase ZO-1, occludin, and claudin-1 expression	Prevent LPS-induced intestinal barrier injury and downregulate TLR4, proinflammatory cytokines (TNF-α, IL-1β, and IL-6).	[52]
*B. longum* subsp. *longum* YS108R	Mouse	DSS colitis	Maintain ZO-1, claudin-1 and mucin-2 expression	Decrease the proinflammatory cytokine IL-6 and IL-17A levels after DSS challenge.	[53]
*Bifidobacterium pseudocatenulatum* MY40C and CCFM68	Mouse	DSS-induced colitis	Increase the localization and expression of β-catenin, claudin-3, and ZO-1	Alleviate DSS-induced colitis and downregulate TNF-α and IL-6, upregulate IL-10 and PPAR-γ, and inhibit TLR4/NF-κB pathway.	[54]
*Bifidobacterium pseudolongum* Bp7 and Bp8	Mouse	DSS colitis	Elevates transcription levels of occludin, claudin-1, and ZO-1 relative to DSS group	Regulates the PPAR-γ/STAT3 pathway (elevate MYD88); anti-inflammatory (reduces colonic TNF-α and IL-1β levels and elevates colonic IL-10 levels).	[55]
Fermentation products *B.* Bb12	Caco-2	—	Increase TER	Protect against deoxycholic acid-disruption of barrier.	[56]
Purified galactooligosaccharide, derived from a mixture produced by the enzymic activity of *B. bifidum*	HT-29 16E; mouse model	*S. typhimurium* infection	Preserve formation of brush borders and tight junctions	Prevent the adherence or invasion of *S. typhimurium* to enterocytes.	[57]
Two strains of *B*. isolated from humans	Germ-free mouse model	Killed *E. coli*, *Klebsiella pneumoniae*, *Yersinia pseudotuberculosis*, *S. aureus,* and *S. typhimurium*	—	—	[58]

Abbreviations: AHR, aryl hydrocarbon receptor; ALT, alanine transaminase; AST, aspartate transaminase; DNBS, dinitrobenzene sulfonic acid; DSS, dextran sodium sulfate; FITC, fluorescein isothiocyanate; HFD, high-fat diet; IBS, irritable bowel syndrome; IFN, interferon; JAM, junctional adhesion molecule; MLCK, myosin light-chain kinase; mTOR, mammalian target of rapamycin; MYD88, myeloid differentiation primary response protein 88; NCRP, native C-reactive protein; NEC, necrotizing enterocolitis; NFκB, nuclear factor kappa-light-chain-enhancer of activated B cells; NOD2, nucleotide-binding oligomerization domain-containing protein 2; NRF, nuclear respiratory factor; PI3K, phosphatidylinositol 3-kinase; PPAR, peroxisome proliferator-activated receptor; TC, total cholesterol; TER, transepithelial resistance; Tff3, trefoil factor 3; TG, triglyceride; TJ, tight junction; TLR, toll-like receptor; TNBS, 2,4,6-trinitrobenzene sulfonic acid; TOLLIP, toll-interacting protein; ZO, zonula occludens.

## *Bifidobacterium* Species and Strains

**FIGURE 2:**
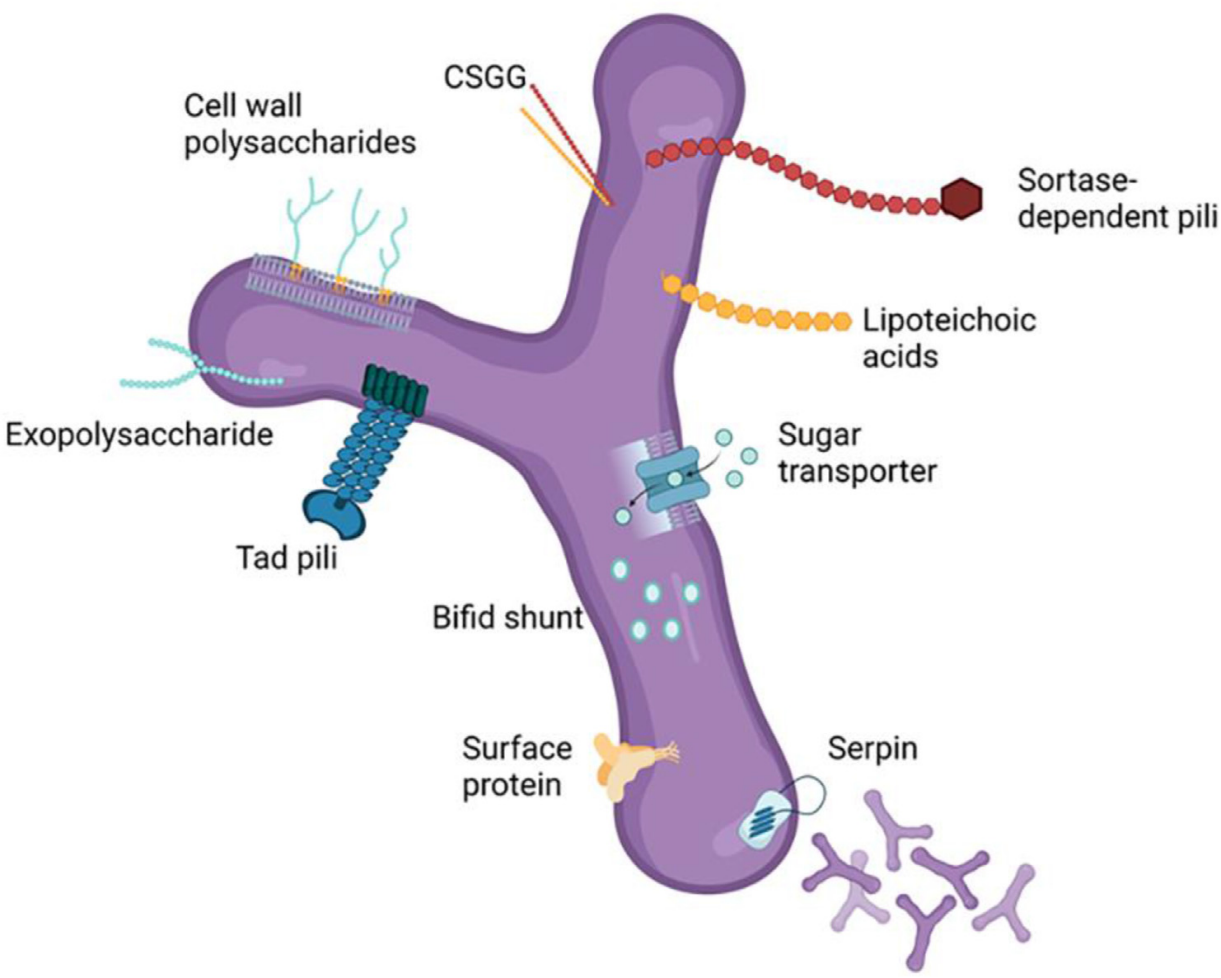
Schematic overview of the bifidobacterial surface structure. The bacterial surface molecules responsible for bifidobacterial-host cross-talk encompass a range of molecules, including exopolysaccharides, cell wall polysaccharides, lipoteichoic acid, surface protein, peptidoglycan and proteins such as CSGG. Abbreviation: CSGG, cell surface β-glucan/galactan. (Created with BioRender).

Among the various probiotic bacteria reported to date, *Bifidobacterium* spp. are one of the most widely studied and utilized probiotic bacteria. The genus *Bifidobacterium* belongs to the phylum Actinobacteria and currently comprises 80 (sub) species, which are distributed across different ecological niches, including the gastrointestinal (GI) tract and oral cavity of humans [59,60]. They are gram-positive, nonmotile, nonsporulating anaerobic bacilli. *Bifidobacterium* spp. are the first to colonize the human intestine, a phenomenon driven by the bifidogenic activities of certain mother milk-derived oligosaccharides [61]. In gram-positive bacteria, the cell wall has unique features; it includes a thick peptidoglycan sacculus with added proteins, lipoteichoic acids, exopolysaccharides, polysaccharides, and cell surface β-glucan/galactan (CSGG). Some species also have an outer layer of proteins arranged in a special pattern called sortease-dependent pili, tight adherence (Tad) pili, or other specific surface proteins (shown in Figure 2). These structures give each bacterium its own distinct properties, which can vary by species and strain. Consequently, they account for 40% to 80% of microorganisms in the intestinal tract of breastfed infants [62,63]. The absence of colonization by *Bifidobacteria* has been found to be associated with the development of allergic disorders in childhood [64]. *B. breve*, *B. bifidum*, *B. longum*, and *B. infantis* are the most commonly detected bacteria at the infant stage, with *B. bifidum* being the most prominent species, followed by *B. breve, B. longum*, and *B. infantis* [26,59]. As age progresses, the overall concentration of *Bifidobacterium* decreases but remains relatively stable (2%–14%) throughout adulthood and decreases again in old age [65]. The most commonly identified species in the adult gut include *B. adolescentis* and *B. catenulatum*, followed by *B. longum* and *B. bifidum* [66,67]. However, there is no absolute infant compared with adult division of bifidobacterial species. Healthy adults generally have a prevalence of *Bifidobacterium* exceeding 90%, with only a small number of species observed per individual [68]. Additionally, this decline in *Bifidobacteria* population has been linked to a decrease in their ability to adhere to the intestinal mucosa. However, it is still uncertain whether this reduced adhesion is caused by changes in the microbiota composition, alterations in the structure of the mucus, or the alteration of TJ barrier permeability [69,70].

**FIGURE 3:**
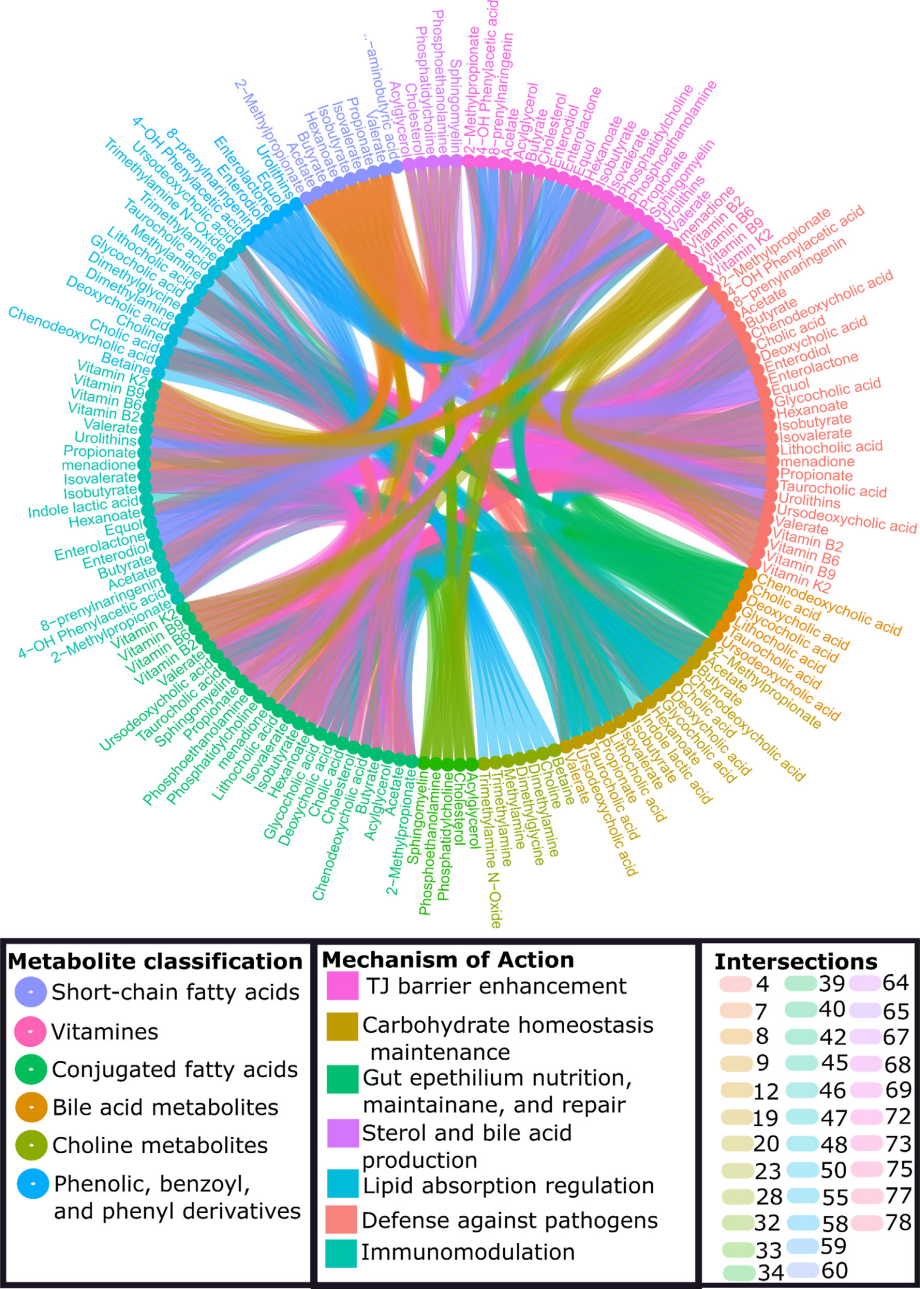
Metabolic capacities of *Bifidobacterium* microbial metabolites are relevant to the microbe-host interaction network. These capacities do not necessarily imply species-specificity or universality among all strains; instead, they suggest a more complex interaction and mechanism involving the metabolites [75,77,79]. Abbreviation: TJ, tight junction. (Created with Setworks v1).

One of the crucial functions of *Bifidobacterium* is its ability to metabolize undigested food residues, such as poly- and oligosaccharides, and utilize host mucins or hyaluronic acid [71]. The carbohydrate metabolism of *Bifidobacterium* is based on a unique pathway called the “bifid shunt” or fructose-6-phosphate shunt [72]. This pathway involves the conversion of fructose-6-phosphate from glucose to erythrose-4-phosphate by the enzyme fructose-6-phosphate phosphoketolase [73,74]. Furthermore, knockouts of the bifid shunt pathway demonstrate that this pathway plays a crucial role in the survival and proliferation of *Bifidobacteria* [75]. As a result, acetate and lactate, which are classified as short-chain fatty acids (SCFAs), are formed (Figure 3) [76]. *Bifidobacteria* exhibit various mechanisms of probiotic action, often with strain-specific characteristics and the secretion of specific metabolites. To provide an overview of the complexities and interactions involved, refer to Figure 3. In addition to SCFAs, *Bifidobacteria* can produce antioxidants, conjugated linoleic acids, polyphenols, vitamins B and K, γ-aminobutyric acid, and a specific type of bacteriocin known as lantibiotics as secondary products [74,[77], [78], [79], [80]], as shown in Figure 3.

*Bifidobacteria* have been commercially exploited as probiotic agents due to their well-established health benefits and “Generally Recognized As Safe” status [81]. The claimed homeostatic and health-promoting activities exerted by *Bifidobacteria* are numerous. These activities include the establishment of a healthy microbiota in preterm infants [53], protection against pathogens [82], enhancement of the intestinal gut barrier [83,42], promotion of an anti-inflammatory environment through modulation of the host immune response [84], production of vitamins and SCFAs, digestion of plant oligo- and polysaccharides, and suppression of the production of potentially toxic and carcinogenic metabolites [58] (Figure 3). As an innate member of the human gut, *Bifidobacterium* has proven to be essential for maintaining intestinal epithelial barrier integrity [56]. Furthermore, in a randomized intervention trial conducted on extremely premature infants, a probiotic mixture consisting of 4 strains of *Bifidobacterium* species and one strain of *Lactobacillus* was administered [46]. The study utilized 16S and internal transcribed spacer rRNA sequencing, as well as metabolomics and cytokine levels in stool, to analyze the effects. The results demonstrated that *Bifidobacterium* strains, but not *Lactobacillus rhamnosus*, were able to effectively establish colonization in the gut of premature infants. Moreover, the maturation of the gut microbiome driven by *Bifidobacterium* strains was associated with a reduction in intestinal inflammation and the promotion of an anti-inflammatory immune environment [46]. Indeed, a recent study cataloging the microbiota of patients with ulcerative colitis (UC) by 16S rRNA microbial profiling revealed a substantial decrease of *Bifidobacteria*, notably *B. bifidum*, suggesting that this taxon plays a biological role in the etiology of UC and also highlighting the importance of *B. bifidum* as a microbial biomarker for UC [67]. Notably, numerous studies spotlight *Bifidobacteria*, with many of them being referenced within this comprehensive review. These studies specifically target strains that are prevalent in both infants and adults, as well as those that exhibit dysregulation in disease states.

## *Bifidobacterium* Regulation of Intestinal TJ Barrier

### Effects of *Bifidobacterium* and TJ barrier in IBD

Previous studies have demonstrated that pretreatment of human intestinal epithelial cell lines (Caco-2, HT-29, and T-84) with *Bifidobacteria* species confers protective effects against TJ barrier impairment induced by various factors [28,43,85]. These protective effects are mediated through upregulation of TJ protein expression (notably occludin and ZO-1). Furthermore, *Bifidobacteria* species also modulate various protein kinase signaling pathways, leading to the phosphorylation of TJ proteins. This phosphorylation can either promote TJ formation or redistribution and complex stabilization [32,33,83]. The beneficial effects of *Bifidobacteria* in treating various GI disorders has also been reported in various mouse models [47]. Bergmann et al. [56] reported the protective effect of *B. infantis* in a mouse model of NEC. Mouse pups administered *B. infantis* exhibited an attenuated increase in intestinal permeability compared to dam-fed controls. The pups also showed preserved occludin and claudin-4 localization at TJs and a decreased incidence of NEC. Additional studies in Caco-2 cells demonstrated a dose-dependent enhancement of the TJ barrier by *B. lactis* 420 supernatant [43]. Another species, *B. bifidum,* has been shown to improve intestinal integrity in a rat model of NEC [41]. To the contrary, *B. animalis* subspecies *lactis* was shown to efficiently restore the gut barrier permeability in a DNBS (dinitrobenzene sulfonic acid)-induced low-grade inflammation model in mice. *B. animalis* subspecies *lactis* protected the intestinal barrier by normalizing the levels of several TJ proteins, particularly claudin-4, and also by restoring the Th1/Th2 ratio balance in colonic goblet cell population [54]. Srutkova et al. [52] showed that *Bifidobacterium* can ameliorate acute dextran sodium sulfate (DSS)-induced colitis in mice. Prophylactic administration of *B. longum* CCM 7952 was capable of preventing the damage by DSS-induced colitis is a strictly strain-specific manner. A recent study by Zhao et al. [38] showed that *B. dentium* N8 offered protective effects [39] in lipopolysaccharide (LPS)-treated Caco-2 cells by increasing the expression of TJ genes ZO-1, occludin, and claudin-1. Additional studies in DSS-induced colitis in mice demonstrated that supplementation with *B*. *pseudocatenulatum* MY40C and CCFM680 strains alleviated DSS-induced colitis by protecting the intestinal barrier, modulating the gut microbiota, suppressing proinflammatory cytokines, and inhibiting the toll-like receptor 4 (TLR4)/nuclear factor kappa-light-chain-enhancer of activated B cells (NF-κB) pathway [39]. These treatments led to increased levels of TJ proteins (ZO-1, β-catenin, and claudin-3) and mucin-2, as well as decreased levels of tumor necrosis factor (TNF)-α and interleukin (IL)-6 while IL-10 and peroxisome proliferator-activated receptor (PPAR)-γ levels were increased [39]. Additionally, they examined how *B*. *pseudocatenulatum* MY40C and CCFM680 strains altered the gut microbiome landscape, finding that both strains increased *Allobaculum* and reduced *Sutterella*, *Bacteroides*, and *Oscillospira* in the gut microbiota [39]. Hsieh et al. [33] demonstrated B. bifidum W1U2 strains can protect the intestinal epithelial barrier from TNF-α-induced disruption and facilitate the maintenance of epithelial TJ integrity. Moreover, the upregulation of SCFA production, particularly acetate and formate, has been identified as a mechanism that contributes to the restoration of the epithelial TJ barrier [33] (Figure 3 depicts a potential metabolite secreted by *Bifidobacterium*). Zhao et al. [34] showed that *B. longum* subsp. K5 strain, but not the K15 strain, has the ability to alleviate the inflammatory response and provide protection against LPS-induced intestinal barrier injury. This effect is achieved by upregulating the mRNA expression levels of ZO-1, occludin, and claudin-1, which are important TJ proteins involved in maintaining barrier integrity. Additionally, the K5 strain downregulated the expression of TLR4, a receptor involved in LPS recognition, as well as the production of proinflammatory cytokines such as TNF-α, IL-1β, and IL-6. In a related study with *B. bifidum* FL-228.1, it was found that this strain protects against DSS-induced intestinal damage in mice by significantly increasing the ratio of IL-10/IL-12, as well as enhancing levels of mucin-2 production and claudin-4 in the colon [86]. A recent investigation involving *B. bifidum* FL-228.1 illustrated that administration of *B. bifidum* FL-228.1 and *B. bifidum* FL-276.1 for 2 wk before DSS induction resulted in more effective alleviation of colitis symptoms compared to intervention after DSS induction began [87]. This protection was attributed to the increased production of indole-3-lactic acid (ILA) by *B. bifidum* FL-228.1 and *B. bifidum* FL-276.1, which regulates the aryl hydrocarbon receptor (AHR)/nuclear respiratory factor 2 (NRF2)/NOD-like receptor family pyrin domain-containing 3 (NLRP3) pathway [87]. Additional studies conducted on Caco-2 cells demonstrated that ILA enhances epithelial barrier function by upregulating ZO-1, occluding, and claudin-4 through AHR activation via the AHR/NRF2/NLRP3 pathway [87]. In a more recent study by Al-Sadi et al. [88], it was demonstrated that *B*. *bifidum* significantly increased intestinal TJ barrier function in cell culture and live mice in a strain-specific manner. These studies also revealed that *B. bifidum* protected against DSS-induced colitis by targeting the intestinal TJ barrier in mice. In addition to live *B. bifidum* providing barrier enhancement, a novel study demonstrated that both *B. bifidum* (BIA-7) and its extracellular vesicles (EVs) can activate distinct signaling pathways [89]. *B. bifidum* was found to activate the Notch-1/Hes-1 pathway, possibly through its interaction with Notch-1 receptors on the cell’s surface and by increasing *AHR* gene expression. However, it was observed that *B. bifidum* alone did not activate the AHR pathway, as evidenced by the absence of increased cytochrome P450 family 1 subfamily A member 1 (CYP1A1) gene expression, which indicates AHR pathway activation [89]. Intriguingly, only the *B. bifidum* EVs were able to penetrate the cells and activate the AHR pathway, leading to significant upregulation of CYP1A1, occludin, and ZO-1 gene expressions. These findings indicate that *B. bifidum* and its derived EVs can activate different signaling pathways, with EVs specifically modulating the gene expression of TJ proteins [89].

### Effect of *Bifidobacterium* and TJ barrier on early-life development and longevity

Kiu et al. [90] aimed to investigate the potential impact of *Bifidobacterium* on the maintenance of IEC homeostasis during the crucial developmental phase of early life. The administration of *B. breve* UCC2003 resulted in significant modifications in the transcriptome of neonatal murine IECs. Approximately 4000 genes were found to be significantly upregulated while around 450 genes were downregulated [90]. The upregulated genes include important ones associated with the functionality of the epithelial barrier, such as ZO-1, ZO-2, ZO-3, occludin, and claudin-12 [90]. Furthermore, a substantial increase in the expression levels of JAM-B and claudin-34c1 was observed [90]. These findings provide further confirmation regarding the critical role of *Bifidobacterium*, as a member of the early-life microbiota, in the development of the intestinal epithelium.

Extended studies conducted in aged mice have provided further evidence supporting the efficacy of administering *L. casei* LC122 and *B. longum* BL986 in exhibiting promising antiaging effects [50]. This treatment regimen has been observed to elicit enhancements in cognitive performance and muscular function while concurrently reducing inflammation and oxidative stress in peripheral tissues. Moreover, it has demonstrated efficacy in ameliorating age-related deterioration of gut barrier function by upregulating the expression of claudin-1, ZO-1, and JAM-A mRNA in both the jejunum and colon [50]. Notably, treatment with *B. longum* BL986 showed a substantial increase in JAM-A expression and a more pronounced anti-inflammatory response compared to the treatment with *L. casei* LC122 [50].

### Effects of *Bifidobacterium* and TJ barrier on liver disease

Numerous studies involving animal models and human patients with alcoholic liver disease and metabolic disorders have shown that prolonged consumption of alcohol and a high-fat diet (HFD) causes an imbalance in gut microbiota and microbial metabolites, leading to defects in the intestinal epithelial barrier [13,50,89,90]. It is hypothesized that this increased gut permeability leads to a higher concentration of LPS in portal blood circulation, where LPS then binds to TLR4 and activates NF-κB, which in turn stimulates the expression of proinflammatory cytokines [39,91]. *Bifidobacterium* has been reported to improve the paracellular permeability in Caco-2 monolayers treated with LPS by significantly decreasing the production of proinflammatory cytokines (such as IL-6 and TNF-α) and upregulating TJ protein (occludin, claudin-3, and ZO-1) expression and localization [32]. Li et al. [40] showed that *B*. *adolescentis* CGMCC 15058 improved liver injury, enhanced the intestinal barrier, and restored the gut microbiota from dysbiosis in D-galactosamine-treated rats. Prior treatment with *B. adolescentis* led to a significant reduction in elevated levels of alanine transaminase and total bile acid while enhancing the expression of mucin-4 and ZO-1. Additional studies in HFD utilizing oral administration of *B. adolescentis* IM38 to mice showed potent suppression of inflammatory markers such as inducible nitric oxide synthase (iNOS), cyclooxygenase 2 (COX-2), TNF-α, and IL-17, which are typically elevated in HFD inflammation. IM38 also reversed the HFD-induced suppression of IL-10 levels in mice. Furthermore, in both HFD-induced obese mice and LPS-stimulated Caco-2 cells, IM38 increased the levels of TJ proteins (ZO-1, occludin, and claudin-1) in the colon [92].

### Effects of *Bifidobacterium* and TJ barrier on RA

It has been observed that patients with RA often have an altered gut microbiota [93]. RA is a chronic inflammatory condition characterized by joint inflammation, swelling, and stiffness [94]. These disturbances in the gut microbiota can potentially lead to damage to the integrity of the intestinal TJ barrier in patients with RA. Li et al. [95] examined the impact of *B*. *breve* CCFM1078 on the intestinal barrier and systemic inflammation in rats with collagen-induced arthritis (CIA). The results revealed that *B. breve* CCFM1078 has the ability to restore the intestinal barrier (via ZO-1, claudin-1, claudin-3, occludin, and JAM-B), decrease the translocation of LPS, regulate the composition of gut microbiota (*B. breve* and *B. pseudolongum* subsp. *pseudolongum*), and increase the levels of SCFAs (acetic acid, propionic acid, butyric acid, and valeric acid) in the intestine. Consequently, CCFM1078 reduced the release of proinflammatory cytokines, regulated immune dysfunction, and inhibited the TLR4/MYD88-dependent pathways and downstream inflammatory pathways, thereby alleviating joint inflammation in CIA rats. These findings indicate that *B. breve* CCFM1078 has the potential to alleviate joint inflammation by modulating the gut microbiota profile and enhancing the intestinal TJ barrier [95].

## Mechanisms of *Bifidobacteria*-Induced Modulation of the Intestinal TJ Barrier

### *Bifidobacterium* adhesion and metabolism in the gut epithelium

Probiotics exhibit strain-specific and even subspecies-specific effects on the intestinal epithelial TJ barrier. Recent research has made significant progress in unraveling the molecular mechanisms underlying the beneficial effects of certain *Bifidobacteria* strains. Comparative genomics has begun to reveal differences in genes associated with host adhesion between various strains and subspecies of *Bifidobacteria* [96]. In a recent comparison of *B. dentium* N8 and E7 (isolated from the feces of breastfed infants), N8 offered significant protective effects against DSS in mouse models and attenuated the LPS-induced increase in paracellular permeability in Caco-2 monolayers, while E7 had no significant effects in either model [38]. Comparative genomics of these strains revealed the presence of genes specific to adhesion ability and immune system regulation in N8 that were absent in the E7 genome [38]. Genes coding for extracellular structures, the main types present in *Bifidobacteria* being Tad pili and sortase-dependent pili (the structure of the Tad pili is depicted in Figure 2), have been identified in several *Bifidobacterium* species, including *B*. *longum* DSM 20219 and *B. bifidum* JCM 1254 [96]. Additionally, the DSM 20219 genome revealed 3 sortase A genes and fimbrial isopeptide formation D2 domain-containing protein, which are also associated with adherence and interaction with host proteins [96]. A recent comparative genomic analysis of commercial multispecies probiotic product VSL#3 found gene clusters coding for Tad pili (shown in Figure 2) in *B. breve* BB02, *B. animalis* subsp. *lactis* BL03 and BI04, structures which are known to maintain intestinal barrier integrity and encourage host–bacteria interaction [97]. Adhesion to the intestinal mucosa is a critical factor for successful colonization and functioning of *Bifidobacteria*. Zhang et al. [35] investigated the impact of different carbon sources, including 2′-fucosyllactose (2′-FL), galactooligosaccharides, and glucose, on the growth and adhesion properties of *B. bifidum* DNG6 to Caco-2 cells. Notably, the utilization of 2′-FL as a carbon source significantly enhanced the adhesion ability of *B. bifidum* DNG6. Additionally, the expression levels of adhesion-associated genes, including transaldolase (TAL), enolase, GroEL, and elongation factor Tu, were markedly higher in *B. bifidum* DNG6 grown in 2′-FL compared to galactooligosaccharides and glucose following incubation with Caco-2 cells [35]. These findings support the potential utilization of 2′-FL as a prebiotic in infant nutritional supplements and underscore the importance of its role as a carbon source, emphasizing its beneficial effects on the adhesion of *Bifidobacteria* and barrier enhancement. Recently, Zhang et al. [98] demonstrated that dietary supplementation with fructooligosaccharide (FOS) in weanling pigs led to notable increases in the mRNA expression levels of important TJ proteins (claudin-1, claudin-2, claudin-4, and occludin) in the ileal mucosa. Additionally, significant upregulation was observed in the mRNA expression of ZO-1, claudin-1, occludin, and pBD-1 within the colonic mucosa upon FOS supplementation [98]. FOS supplementation improved the expression of TJ proteins in weanling pigs, possibly linked to increased concentrations of lactic acid and acetic acid, which decrease pH in the ileal digesta, thereby promoting a healthier intestinal microenvironment [98].

### *Bifidobacterium*-mediated effects on the TLR2 signaling pathway

**FIGURE 4:**
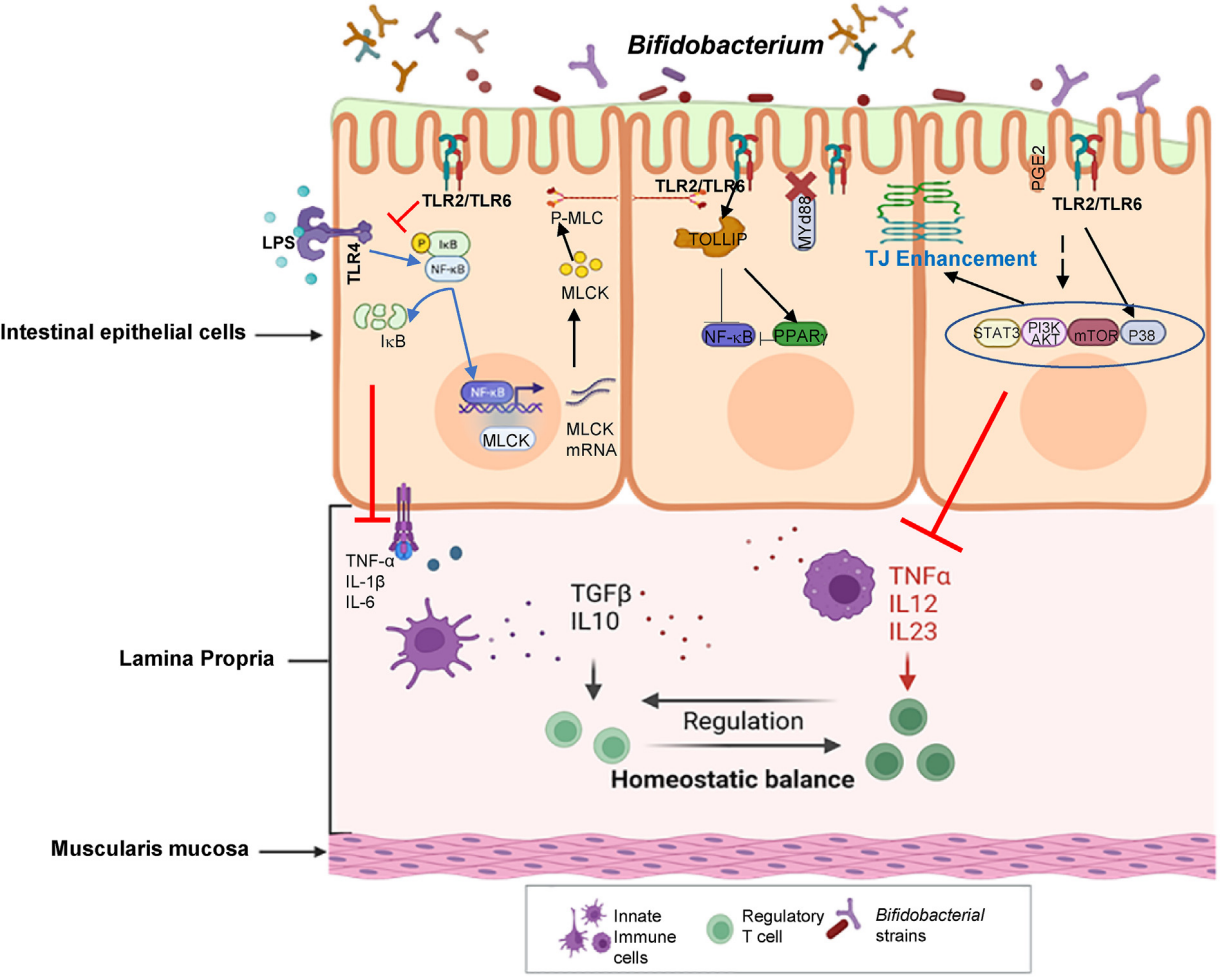
Schematic representation of *Bifidobacteria* targeting TJ barrier function via TLRs signaling pathways. Abbreviations: IκB, inhibitor of nuclear factor kappa B; MLCK, myosin light-chain kinase; MYD88, myeloid differentiation primary response protein 88; NFκB, nuclear factor kappa-light-chain-enhancer of activated B cells; PGE2, prostaglandin E2, PI3K, phosphatidylinositol 3-kinase; P-MLC, phosphorylated myosin light chain; PPAR-γ, peroxisome proliferator-activated receptor gamma; TGF, transforming growth factor; TLR, toll-like receptor, TNFR, tumor necrosis factor receptor; TOLLIP, toll-interacting protein. (Created with BioRender).

Previous studies of probiotics known to exert beneficial effects on the intestinal barrier, including *Lactobacillus acidophilus* [99], *Akkermansia muciniphila* [74], and Bacteroides fragilis [75], have identified TLR2 as a crucial pathway in mediating this host–bacterial interaction. Khailova et al. [41] found that oral administration of *B. bifidum* to rat pups activates TLR2 signaling, increases COX-2 expression, leads to elevated production of prostaglandin E2 in the ileum, and provides protection against intestinal apoptosis associated with NEC. Similarly, a recent study identified a specific strain of *B. bifidium* (BB1) that caused an enhancement of the intestinal epithelial TJ barrier both in vivo and in vitro and found that attachment to TLR2 at the apical membrane of enterocytes was the main mechanism of action [88]. Additionally, this binding was found to be mediated by the heterodimerization of TLR2 with TLR6 but not TLR1 [88]. The nature of the host–bacteria interaction in 2 subspecies of *B. longum* (BI 371 and BI 7952) was also found to involve signaling through TLR2, although only BI 7952 showed a protective effect on the intestinal epithelial barrier [52]. Interestingly, both of these strains were found to also contain ligands for nucleotide-binding oligomerization domain-containing protein 2 (NOD2), although the nature of this interaction and downstream pathways have yet to be investigated [52]. Further studies by Abdulqadir et al. [44] demonstrated that *B. bifidum* strain BB1, but not BB4, is responsible for strengthening the intestinal epithelial TJ barrier. This enhancement is achieved through an increase in toll-interacting protein (TOLLIP) mediated by TLR2 in a manner independent of MYD88 (myeloid differentiation primary response 88) [44,49]. TOLLIP is a powerful inhibitory cytosolic adaptor protein that suppresses MYD88 activity. The observed enhancement in the TJ barrier caused by BB1 was abolished when TLR2 and TOLLIP were specifically silenced through targeted knockdown [44]. The specific components of *B. bifidum* that are responsible for enhancing the TJ barrier and their interactions with host cells have not been determined conclusively. In a study by Verma et al. [29], it was found that CSGG (the structure of CSGG is depicted in Figure 2) derived from *B. bifidum* plays a crucial role in suppressing intestinal inflammation. These CSGG molecules induce the generation of regulatory T cells (Foxp3^+^ T cells), which help regulate the immune response. Furthermore, CSGG stimulates the production of regulatory dendritic cells (DCs) through a mechanism that involves TLR2. This suggests that CSGG-mediated induction of Treg cells occurs through a TLR2-dependent process involving regulatory DCs [29]. In a subsequent study, cell surface polysaccharides were isolated from *B. bifidum* strain PRI1 [37]. The results demonstrated that this bacterium produces a complex mixture of polysaccharides that can be classified into 2 main groups: phospho-glycero-β-galactofuranan (PGβG) and a mixture of 4 neutral polysaccharides known as CSGG. The CSGG fraction is composed of β-(1 → 6)-glucan, β-(1 → 4)-galactan, β-(1 → 6)-galactan, β-galactofuranan, and starch. These 2 fractions exhibited distinct immune responses when tested on DCs. PGβG stimulated proinflammatory immune responses by increasing interferon-γ levels, whereas CSGG induced immunosuppressive regulatory T cells and IL-10 production [37]. An overview of *Bifidobacteria*’s effect on TLR2 and these related pathways is shown in Figure 4.

### *Bifidobacterium*’s role in modulating the NF-κB and MAP kinase pathway

The 2 known downstream pathways primarily involved in regulating the TJ barrier include NF-κB and MAP kinase [88]. Al-Sadi et al. [88] found that the protective effect of BB1 was mediated by the activation of p38 kinase and was independent of the NF-κB signaling pathway. Consistent with these data, Guo et al. [51] showed that *B. infantis* conditioned media inhibited the IL-1β induced NF-κB p65 activation in Caco-2 cells by normalizing the protein expression of occludin and claudin-1. Additionally, Jang et al. [100] showed that the strain-specific *B. longum* LC67 prevented NF-κB activation, transforming growth factor-β-activated kinase 1, and IκBα (inhibitor of nuclear factor kappa B, alpha) phosphorylation, as well as COX-2 and iNOS expression in a 2,4,6-trinitrobenzene sulfonic acid (TNBS)-induced colitis mouse model and restored expression of TJ proteins(occludin, ZO-1, and claudins). Furthermore, treatment with *Bifidobacterium* has a protective effect on the inflammatory response induced by TNF-α in Caco-2 cells. This effect is achieved through the inactivation of NF-κB and p38 kinase pathways [55]. Inhibition of NF-κB activation is a commonly observed mechanism of action of *Bifidobacteria*. Yue et al. [101] investigated the effects of combining *B. bifidum* E3 with *B. longum* subsp. *infantis* E4 (BLBB) in mice treated with LPS. They found that this combination inhibited the TLR4/NF-κB and mitogen activated protein kinase (MAPK) signaling pathways. Additionally, there was an increase in the number of IgA^+^ plasma cells, CD4^+^/CD8^+^ T cells, and DCs in the BLBB group [101]. The levels of diamine oxidase and D-lactic acid in the serum were reduced in the BLBB group after LPS injection compared to the LPS alone group. Furthermore, the BLBB treated group also showed increased expression levels of TJ proteins (ZO-1, occludin, and claudin-1), mucin-2 mRNA, and decreased expression levels of inflammatory cytokines (IL-1β, IL-6, and TNF-α) [101]. Guan et al. [102] demonstrated that *B. longum* K2-21-4 exhibits a protective effect against LPS-induced injury. *B. longum* K2-21-4 enhanced cellular proliferation, boosted overall cellular protein levels, accelerated the cell cycle, diminished apoptosis, and heightened the activity of ATPases, as well as aspartate aminotransferase and alanine aminotransferase, in LPS-induced injury. Additionally, this protective effect was achieved through the activation of 2 signaling pathways, namely the Wnt/β-Catenin pathway and the phosphatidylinositol 3-kinase/Akt/mammalian target of rapamycin pathway. This study showed that activated *B. longum* K2-21-4 enhances the TJ barrier of ZO-1, occludin, and claudin-1, further contributing to its protective properties against LPS-induced injury in CCD841 CoN cells [102]. In similar studies, Shang et al. [103] demonstrated the effective alleviation of DSS-induced colitis pathogenesis in mice and the mitigation of LPS-induced disruption in CCD-841 CoN cells using *B. bifidum* H3-R2. This protective effect was attributed to the treatment with recombinant GroEL and TAL proteins of *B. bifidum* H3-R2, which facilitated the upregulation of TJ proteins (ZO-1, occludin, and claudin-1) in the context of LPS-induced inflammation [103]. Furthermore, in-depth analyses indicated that these proteins may exert their beneficial effects through the inhibition of NF-κB, MLCK, RhoA/Rho-associated protein kinase, and MAPK pathways [103].

### *Bifidobacterium*’s role in modulating the PPAR-γ/Signal transducer and activator of transcription 3 (STAT3) pathway

Another important pathway for enhancing and repairing the intestinal barrier is the PPAR-γ/STAT3 pathway. In a study by Guo et al. [104], *B. pseudolongum* strains Bp7 and Bp8 were found to ameliorate intestinal barrier function in DSS colitis mice via increased expression and activation of the PPAR-γ/STAT3 pathway. Previous studies have also shown that probiotics influence bacteria–host interactions by modulating PPAR-γ [36,[105], [106], [107]]. Nepelska et al. [107] examined conditioned media from 57 commensal gut bacterial strains, including 23 species of *Bifidobacteria,* to modulate PPAR-γ activities. They linked transcriptional activity of PPAR-γ to the presence of butyrate and propionate, 2 of the main metabolites of intestinal bacteria. Only 2 species of probiotics (*A. parvulum* and *P. copri)* were found to induce the expression of PPAR-γ genes, including adipose differentiation-related protein and angiopoietin-like protein 4 in HT-29 cells. Another study showed a protective effect of live *B. bifidum* against IL-1β induced increase in intestinal permeability in vitro and in vivo. The strain’s specific protective effect of live *B. bifidum* against the IL-1β induced increase in intestinal TJ permeability was mediated by TLR2/PPAR-γ activation, dependent inhibition of NF-κB, and MLCK gene activation [108]. Interestingly, Abdulqadir et al. [49] showed that in administration of 5 live strains of *B. bifidum* to Caco-2 cells, only 1 strain (BB1) activated PPAR-γ, leading to a cyto-to-nuclear translocation. Additionally, pretreatment with BB1 inhibited TNF-α-induced increase in transepithelial electrical resistance (TER) in Caco-2 cells and prevented the TNF-α-induced activation of NF-κB in a PPAR-γ dependent manner [49,109]. Probiotics are known to activate STAT3 by activating anti-inflammatory factors, maintaining gut integrity, and regenerating the epithelium in the intestinal crypts [[110], [111], [112], [113]]. Sonicated *Lactobacillus* spp., *Bifidobacterium* spp., and a mixed cocktail showed significant anti-inflammatory effects on HT-29 cells [110]. This was achieved by downregulating certain genes in the NF-κB pathway, including Janus kinase, Toll-interleukin-1 receptor domain-containing adaptor protein, interleukin-1 receptor-associated kinase 4, NF-κB essential modulator, and ribosomeinactivating protein [110]. Additionally, *Bifidobacterium* spp. treatment enhanced STAT3 signaling and led to reduced production of IL-6 and IL-1β, further supporting the anti-inflammatory response [110].

Based on the presented research, it is likely that different species and strains of *Bifidobacteria* will have varying effects on the enhancement of the intestinal TJ barrier (as shown in Figure 4) and the degree of intestinal anti-inflammatory response. Therefore, it is important to acknowledge *Bifidobacteria*’s role in the intestinal TJ barrier as a fundamental target for therapy in treating IBD, NEC, and other GI-related disease.

## The Role of *Bifidobacteria* in Therapeutically Targeting the Intestinal TJ Barrier and Microbiome in Disease

Results obtained from various human clinical trials also agree on the beneficial effects of *Bifidobacterium* in maintaining and treating intestinal epithelial barrier. For example, the administration of *B. longum* subsp. *infantis* CECT 7210 and *B. breve* K-110 inhibited rotavirus-induced sporadic diarrhea in infants [114]. A recent study by Patole et al. [115] reported that routine administration of *B. breve* M-16V lowered the incidence of NEC in neonates born before 34 wk gestation. Another double-blind study showed a significant reduction in antibiotic-associated diarrhea in infants treated with a commercial probiotic formula containing *B. bifidum* and *Streptococcus thermophiles* [116]. *B. animalis* subsp. *lactis* BB12 helps maintain fecal acetate levels in subjects receiving antibiotics, which is necessary to prevent postantibiotic dysbiosis [48]. Additionally, in a study involving 113 preterm infants who were exclusively breastfed, it was observed that there was a significant increase in *B*. *breve* strains. These strains are characterized by their exceptional ability to metabolize carbohydrates, which is essential for the development and maturation of the intestinal barrier. This process is important in preventing increased intestinal permeability and NEC [117].

Moreover, a study carried out in patients with UC in Japan strongly demonstrated that administration of *B. longum* BB536, isolated from the feces of a healthy infant, was well tolerated and effective in preventing remission [118]. Previous studies have demonstrated that the viable strain of *B. bifidum* MIMBb75 significantly alleviates symptoms of irritable bowel syndrome (IBS) [119,120]. However, it remains uncertain whether the nonviable form of *B. bifidum* MIMBb75 exerts a significant impact on IBS. In a recent randomized, double-blind, placebo-controlled clinical trial involving 443 participants (221 receiving the heat-inactivated bacterial strain of *B. bifidum* HI-MIMBb75 and 222 receiving placebo), significant alleviation of IBS and its symptoms was observed in the group administered [121]. Additionally, certain *Bifidobacteria* probiotics have the potential to decrease the accumulation of fat in the host. A study conducted using heat-killed *B. lactis* CECT8145 showed a notable increase in lean mass and an improvement in metabolic syndrome among cafeteria-fed obese rats [122]. This finding was further supported by similar studies conducted on abdominally obese individuals using the same probiotic. These studies demonstrated that both live and heat-treated forms of *B. lactis* CECT8145 can effectively reduce anthropometric adiposity biomarkers associated with alterations in the regulation of the host immune system while also promoting the enrichment of the *Akkermansia* genus in the gut of individuals with abdominal obesity [123]. Upon conducting sex-specific analyses, statistical significance was observed exclusively among females [123]. Furthermore, a randomized, double-blind, placebo-controlled trial demonstrated that the administration of the Lab4P consortium of probiotics, which consists of 2 strains of *Lactobacilli* and 2 strains of *Bifidobacteria*, resulted in significant reductions in body weight, BMI, and waist-to-hip ratio (W/H ratio) circumference. The trial also revealed decreases in small dense LDL cholesterol levels overall and improvements in overall well-being [45]. In contrast, the efficacy of one strain of *B. longum* APC1472 in addressing obesity only partially translates from mice studies to humans. In HFD-fed mice, supplementation of *B. longum* APC1472 was linked to reduced body weight, decreased accumulation of fat deposits, and improved glucose tolerance [124]. However, in overweight/obese adults, the supplementation of *B. longum* APC1472 strain did not yield significant changes in the primary outcomes of BMI or W/H ratio. Nevertheless, a positive impact was observed in secondary outcomes of reduced fasting blood glucose levels, cortisol awakening responses and increased active ghrelin in healthy obese adults [124]. Moreover, Niu et al. [57] demonstrated that a higher dosage of *B. animalis* subsp. *lactis* (1 × 10^10^ CFU/kg b.w.) provided significant protection to HFD-induced mice. This protection extended to mitigating body-weight gain, reducing elevated fat percentage, and alleviating dyslipidemia. Additionally, the higher dosage of *B.animals* subsp. *lactis* exhibited remarkable efficacy in safeguarding against increased levels of LPS or endotoxemia [57]. It is important to note that this study has not been replicated in humans.

However, there is evidence that show the effect of *Bifidobacterium* is strain-specific and hence, the interaction of different *Bifidobacterium* species with host cells may have distinct effects [125]. For instance, supplementation of *B. longum* showed a decrease in the expression of genes encoding proinflammatory cytokines [42], while ingestion of *B. animalis* subsp. *lactis* caused an increase in the anti-inflammatory cytokine IFN-α and phagocytic activity [82]. These studies caution that the beneficial effects of one probiotic strain cannot be applied to other species or even subspecies of the same genus.

A major concern regarding the use of probiotics in treating infections is the potential side effects caused by the ingestion of large bacterial loads in sick or immunocompromised patients. One approach to address this concern is to use isolated probiotic-derived bioactive factors that retain the functions of live bacteria. Some studies have suggested that secreted products from specific probiotics alleviate disruptions in intestinal barrier function associated with intestinal disease. Multiple in vitro studies found that bacteria-free conditioned media from *B. infantis* demonstrated several beneficial effects [126]. It increased the TER, reduced the levels of claudin-2, and increased the expression of ZO-1 and occludin. These effects were accompanied by increased levels of phosphorylated extracellular-signal regulated kinase and decreased levels of phosphorylated p38 in T-84 cells [126]. Furthermore, bacteria-free conditioned media of *B. infantis* was found to protect against NEC by suppressing the activation of NF-κB via preserved IκB expression. The conditioned media also suppressed the production of the proinflammatory cytokine TNF-α, a downstream target of the NF-κB-pathway [127] and prevented the intestinal permeability damage caused by IL-1β [51]. Purified galactooligosaccharide, derived by the galactosyltransferase activity of *B. bifidum* (using lactose as the substrate) was shown to reduce the adhesion and invasion of *Salmonella enterica serovar* and *S. typhimurium* both in vitro and in vivo [128]. Furthermore, it was found that the fermentation products of *B.Bb12* protect against deoxycholic acid-induced disruption of the barrier and enhance the intestinal barrier, as measured by TER in Caco-2 cells [28].

The majority of studies involving *Bifidobacteria*-derived bioactives have only been demonstrated in laboratory models. More studies are ongoing to progress to the translational stage and ultimately to clinical trials. Another promising method is the use of engineered *Bifidobacteria* that have been modified to perform a therapeutic function. Numerous clinical trials are currently underway, investigating the potential of engineered probiotics in the treatment of IBD, exemplified by studies like *E. coli Nissle* 1917 (NCT04969679) and *B. breve* Bif195 (NCT04842149) [129,130]. In addition, ActoGenix has pioneered the development of 2 engineered *L. lactis*-based products designed to address IBD, both of which have advanced to the clinical trial phase. The first, AG011, features an engineered *L. lactis* strain capable of secreting human IL-10 and has demonstrated safety and excellent tolerance in a cohort of patients with UC (NCT00729872) [25,130,131]. Meanwhile, AG014, built upon the same *L. lactis* platform as AG011, has been engineered to produce an anti-TNF-α antibody fragment similar to certolizumab [132]. Although AG014 underwent evaluation in a phase I clinical trial, it has not progressed further [130,132,133]. Ongoing in vitro studies are conducting similar investigations on the use of *Bifidobacteria* [134], and future research endeavors will delve into the potential role of *Bifidobacteria* in the treatment of IBD without the side effect of ingesting large bacterial loads in sick or immunocompromised patients.

## Conclusion and Future Perspectives

Although a plethora of studies have demonstrated the health-promoting activities of *Bifidobacteria* species, particularly their role in maintaining the intestinal epithelial TJ barrier, the underlying molecular mechanisms still remain largely unknown. Major challenges behind this include: *1*) the strain-specific activity of *Bifidobacterium,* which makes it difficult to define a specific pathway of action; *2*) the complex interaction of *Bifidobacteria* with human host cells and other members of the gut; and *3*) the notoriously recalcitrant nature of *Bifidobacteria* to genetic modification. The development of effective molecular tools and more focused studies is expected to unravel molecular mechanisms that explain how *Bifidobacteria* interact with human host cells and exert their beneficial effect.

## Author contributions

The authors’ responsibilities were as follows—RA, RA-S: conceptualized and designed the review; RA, J.E: wrote the draft manuscript; RA: prepared figures; RA, JE, RA-S: revised the first draft; and all authors: read and approved the final manuscript.

## Funding

This research was funded by Penn State Department of Medicine.

## Conflict of interest

The authors report no conflict of interest.
